# The Effect of Salary Compensation for Time Spent Teaching in an Orthopaedic Residency Program: An Analysis of Teaching Performance Reviews

**DOI:** 10.5435/JAAOSGlobal-D-21-00307

**Published:** 2022-01-07

**Authors:** Louis C. Grandizio, Eugene P. Warnick, Max D. Gehrman, Joel C. Klena

**Affiliations:** From the Geisinger Medical Center, Department of Orthopaedic Surgery, Danville, PA (Dr. Grandizio), and the Geisinger Commonwealth School of Medicine, Geisinger Musculoskeletal Institute, Danville, PA (Dr. Warnick, Dr. Gehrman, and Dr. Klena).

## Abstract

**Introduction:**

Although there has been a recent emphasis on standardized resident assessments within Accrediation Council for Graduate Medical Education programs, assessments of faculty teaching performance and effectiveness are less frequent. Our purpose was to compare the teaching performance of orthopaedic surgery faculty receiving compensation for time spent teaching with faculty without compensation.

**Methods:**

For this prospective investigation, we collected anonymous resident reviews of 23 orthopaedic faculty within a rural, academic orthopaedic residency program over 2 academic years. Performance reviews of the faculty used a validated assessment of clinical teaching effectiveness with nine domains (faculty knowledge, organization, enthusiasm, rapport, involvement in learning experiences, feedback, clinical skill, accessibility, and overall effectiveness). A composite teaching effectiveness score was determined by adding each of the scores from the individual domains. We compared reviews for faculty members with and without compensation for time spent teaching.

**Results:**

A total of 202 performance reviews for 23 orthopaedic faculty were analyzed. Most of the faculty were male (91%), and 61% received compensation for teaching. No demographic differences were observed between the two faculty groups. Notable differences between the groups were noted in three domains: enthusiasm, ability to establish rapport as well as direction, and feedback. Faculty compensated for teaching demonstrated a markedly higher composite teaching effectiveness score than those without compensation.

**Discussion:**

These data suggest that orthopaedic faculty compensated for teaching responsibilities provide a better educational experience for resident trainees compared with faculty without compensation for teaching. Future studies should aim to assess varying compensation models for teaching responsibilities across different departments.

Graduate medical education relies on an apprenticeship-style model whereby trainees work under the supervision of established physicians.^1^ Effective clinical teaching, particularly within a surgical subspecialty, requires substantial effort and time on behalf of the educator. Careers in academic surgical fields have been described as a tripod with a balance of direct patient care, academic research, and trainee (resident) education. Because of procedural reimbursement decreases and healthcare systems experience continued financial stress, orthopaedic surgeons will increasingly be pressured toward clinical production as a response to this challenging fiscal environment.^2,3^ These financial pressures can function to deemphasize nonrevenue generating activities, such as education and teaching. Compensating orthopaedic surgeons for nonrevenue generating activities varies substantially across departments.^4^ It is clear, however, that the amount of time and effort that physicians put into educating trainees is not reflected in compensation.^5^

For orthopaedic surgeons with teaching and research responsibilities, departments can compensate faculty for these activities directly (with additional salary) or indirectly (with dedicated research/teaching time). In direct compensation models, both teaching and nonteaching faculty have the same clinical productivity expectations, but faculty who teach residents are given additional salary. In indirect compensation models, teaching faculty have reduced clinical productivity expectations but remain with the same salary as their nonteaching peers. Departments that quantify clinical production with work relative value units (wRVUs) and set wRVU benchmarks based on the clinical full-time equivalent (FTE) status may compensate surgeons for nonrevenue generating activities with RVU or FTE reductions. Within their specialty-specific program requirements, the ACGME notes that for orthopaedic residencies, the program director at a minimum “must be provided with the salary support required to devote 20 percent FTE of nonclinical time to the administration of the program”.^6^ However, compensation standards for additional faculty vary by department.

In addition to the variability relative to compensating orthopaedic surgeons for teaching/residency activities, assessing teaching effectiveness also remains difficult. There has been substantial effort over the past decade to improve and standardize both resident education and resident evaluations, as evidenced by the Milestones Program within the ACGME. The questions of how clinical educators should be evaluated and who should do it remain unanswered. Annual performance reviews mandated by the ACGME must include “a review of the faculty member's clinical teaching abilities”.^7^ These faculty reviews have been infrequently analyzed within orthopaedic surgery, and there is a paucity of literature analyzing the relationship between compensation for time spent teaching and teaching effectiveness.

The purpose of this investigation was to compare the teaching performance of orthopaedic surgery faculty receiving compensation for time spent teaching residents with faculty without compensation. We aimed to test the null hypothesis that there would be no difference in performance between these two groups as measured by anonymous resident reviews of teaching performance.

## Methods

Institutional Review Board approval was obtained for this prospective, single-center investigation. This study analyzed the performance reviews of 23 orthopaedic surgery residency faculty over 2 academic years (2018 to 2019 and 2019 to 2020). There were 18 orthopaedic surgery residents during the 2018 to 2019 academic year and 19 residents during the 2019 to 2020 academic year. Our orthopaedic surgery residency is part of a rural academic, level I trauma center in the northeastern United States that functions as a tertiary referral center. The orthopaedic surgeons at our institution receive a base salary for their position commensurate with the sum of their total FTEs (clinical + teaching). Each surgeon is asked to meet a baseline annual wRVU level specific to their subspecialty, which is established based on a 1.0 clinical FTE schedule (100% clinical). Surgeons with 0.8 clinical FTEs and 0.2 teaching FTEs (80% clinical schedule) still receive their full salary, but their RVU benchmark is lowered by 20%.

Dedicated FTEs for teaching are assigned by the orthopaedic residency director. FTE allocations are based on meeting the specific criteria for educational participation. The criteria begin with having a role in direct resident teaching. In addition, FTEs are assigned to faculty with additional responsibilities, such as resident oversight, resident wellness, and faculty development. In addition, each subspecialty has a faculty member designated as director of that subspecialty's education and curriculum, and they are given teaching FTEs. Both the residency program director and the associate program director are assigned teaching FTEs as outlined by the ACGME. All faculty included in this investigation work in full-time positions, and their practices were primarily located at our main clinical campus.

Residents completed their anonymous faculty evaluations over the course of the 2 academic years. Residents were asked only to complete evaluations for faculty members for which they had sufficient contact to evaluate each of the teaching domains. We used a 9-item teaching assessment form designed by Irby.^8^ This validated instrument was designed to assess teaching effectiveness in clinical programs.^8,9,10,11^ Each of the nine domains (faculty knowledge, organization, enthusiasm, rapport, involvement in learning experiences, feedback, clinical skill, accessibility, and overall teaching effectiveness) are scored on a 7-point scale (0 to 6). A higher score indicates better performance. This tool has been demonstrated to be a reliable measure of evaluation in the clinical setting and has been used to validate other assessment tools.^9,10,11,12,13^ In addition to recording the individual scores for each domain, we recorded a composite score, which represented the sum total of the nine individual domain scores.

### Sample Size Calculation

Using a previous study by Irby et al^9^ analyzing teaching performance assessed by trainees, we performed an a priori sample size calculation. For that study, the mean score within the “overall teaching effectiveness” domain was 5.10 with an SD of 1.03.^9^ We considered a clinically notable improvement in teaching effectiveness to be an increase of 10% on the Irby assessment. Using alpha = 0.05, power = 80%, and assuming a 2:1 ratio between the groups, we determined that a minimum of 96 and 48 reviews would be needed for group 1 (faculty with teaching FTEs) and group 2 (faculty without teaching FTEs), respectively.

### Statistics

To compare assessment scores between faculty members who received FTEs and those who did not, we recorded baseline demographics for orthopaedic faculty members within the residency program. We recorded sex, the number of assessments for each faculty member, the number of years that they had been in practice, and whether they received dedicated teaching FTEs. Descriptive statistics were used for reporting the general demographics of our sample of orthopaedic surgery residency faculty. Continuous variables were summarized using ranges, means, and standard deviations. Categorical variables were summarized using frequency and percentages. Comparisons between the two groups were done using the chi-square test for categorical data and the Student *t*-test for normally distributed continuous data. The composition of faculty for sex between the two groups was evaluated with a Fisher exact test. Differences of *P* < 0.05 were considered statistically significant.

## Results

Over the course of the 2 academic years, a total of 202 performance reviews of the 23 orthopaedic surgery faculty were provided by the residents. Table 1 provides demographic information related to the faculty members. Overall, 9% of faculty members were women, and 61% received dedicated teaching FTEs. No demographic differences were observed between the two faculty groups, as noted in Table 1.

**Table 1:** Demographic Comparison of Orthopaedic Surgery Residency Faculty Members With and Without Dedicated Teaching FTEs

Variable	Total	FTE	No FTE	*P*
Faculty members, N (%)	23	14 (61%)	9(39%)	—
Assessments, N (%)	202	131 (65%)	71 (35%)	—
Male faculty, N(%)	21 (91%)	12 (86%)	9 (100%)	0.5020
Years in practice, mean (range)	16 (1-41)	15 (3-41)	19 (1-30)	0.3536

FTE = full-time equivalent

**Figure 1:**
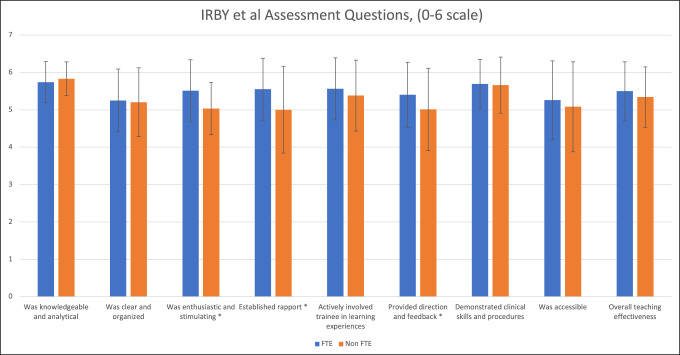
Bar graph depicting the results of faculty assessments completed by orthopaedic surgery residents with comparisons between faculty with and without compensation for teaching. Error bars indicate the SD.

Table 2 summarizes the results of the faculty assessments completed by orthopaedic surgery residents using the Irby instrument. Faculty members with compensation for teaching demonstrated significantly higher scores in the following three domains: “was enthusiastic and stimulating” (*P* < 0.0001), “established rapport” (*P* = 0.0001), and “provided direction and feedback” (*P* = 0.0071), as shown in Figure 1. Overall, the faculty who received dedicated teaching FTEs received a higher composite score compared with the faculty without teaching compensation (49.78 versus 47.54, *P* = 0.0267).

**Table 2:** Results of the Faculty Assessments Completed by Orthopaedic Surgery Residents With Comparisons Between Faculty Members With and Without Teaching FTEs

Variable	Total	FTE	No FTE	*P*
Faculty members, N (%)	23	14 (61%)	9(39%)	—
Assessments, N (%)	202	131 (65%)	71 (35%)	—
IRBY et al assessment questions, (0-6 scale)				
Was knowledgeable and analytical, mean (SD)	5.77 (0.52)	5.74 (0.55)	5.83 (0.45)	0.2348
Was clear and organized, mean (SD)	5.23 (0.87)	5.25 (0.84)	5.20 (0.92)	0.6705
Was enthusiastic and stimulating, mean (SD)	5.34 (0.81)	5.51 (0.83)	5.03 (0.70)	<0.0001
Established rapport, mean (SD)	5.36 (0.99)	5.55 (0.83)	5.00 (1.16)	0.0001
Actively involved trainee in learning experiences, mean (SD)	5.50 (0.88)	5.56 (0.83)	5.38 (0.95)	0.1714
Provided direction and feedback, mean (SD)	5.26 (0.97)	5.40 (0.87)	5.01 (1.10)	0.0071
Demonstrated clinical skills and procedures, mean (SD)	5.68 (0.69)	5.69 (0.66)	5.66 (0.75)	0.8065
Was accessible, mean (SD)	5.20 (1.11)	5.26 (1.05)	5.08 (1.20)	0.2842
Overall teaching effectiveness, mean (SD)	5.45 (0.79)	5.50 (0.78)	5.34 (0.81)	0.1557
Composite score, mean (SD)	48.78 (5.9)	49.46 (5.76)	47.54 (5.99)	0.0267

FTE = full-time equivalent

## Discussion

There is a paucity of previous studies analyzing the relationship between compensation for teaching and teaching performance for faculty within orthopaedic surgery residency programs. In a large, cross-sectional survey of multiple specialties, clinical educators who dedicated more time to teaching and attended faculty development programs focused on teaching were more highly rated by trainees.^14^ In other surveys of nonorthopaedic programs, nonmodifiable faculty factors, such as female sex and personality traits (less extraversion and more openness), were associated with worse teaching performance scores.^15,16^ Although some aspects of faculty reviews by residents may be subject to bias and nonmodifiable factors, our results suggest that decreased clinical productivity expectations (and thus more time to teach) are associated with improved scores on teaching assessments.

Our results suggest that having dedicated time to teach (or incentivizing teaching) is associated with higher assessment scores on teaching performance reviews. Given the lack of previous studies within orthopaedics to directly compare these data, comparisons with departmental incentivization of research help contextualize our findings. Within a single, large surgical department, the development of a self-reported academic RVU system was associated with increases in overall academic productivity and allowed for more equitable distribution of financial compensation for this nonrevenue generating activity.^17^ Even without direct financial compensation, orthopaedic departments have increased academic productivity by restructuring their research programs and allowing for dedicated research time for both residents and faculty.^18^ Although teaching performance is less quantifiable than academic productivity, in this context, it seems likely that increasing protected time or appropriately compensating faculty for teaching would be associated with improvements in teaching performance. We note that some domains within the Irby assessment that did differ between groups (knowledge, clinical skill, and organization) may be more inherent traits and less modifiable, regardless of the compensation strategy. In this context, areas such as providing feedback to trainees, enthusiasm for teaching, and rapport with trainees seem more likely to be influenced by time specifically allocated to teaching.

This investigation has a number of limitations. These data indicate an association, rather than causation, between incentivized teaching and improved performance. There may be confounding bias, and it remains possible that faculty receiving FTEs may have demonstrated higher performance scores even in the absence of compensation for teaching. Although statistically significant, the overall differences in the composite scores between groups were relatively small and it remains unknown how large of a change would be considered educationally impactful. It is likely that institutions differ for academic culture and the value placed on academic contributions. Because these data are from a single, rural academic center in the northeastern United States, it remains uncertain if our findings are generalizable to other institutions. Although we used a validated assessment of teaching performance, it is uncertain if resident assessments of teaching performance are the most effective means of faculty evaluation and introduce the possibility of bias. RVU data were not available for faculty members, and as a result, we were unable to compare clinical production between faculty with and without FTE support. In addition, there are a variety of ways to compensate clinical faculty for teaching responsibilities and it remains uncertain which strategy is optimal. Future investigations should aim to incorporate multiple programs, practice structures, and compensation models.

Compared with orthopaedic faculty who did not receive compensation for teaching, faculty who received compensation scored higher in three domains of the Irby instrument (“enthusiasm”, “establishing rapport”, and “providing direction and feedback”). In addition, compensated faculty demonstrated a markedly higher mean composite score. These data suggest that orthopaedic faculty compensated for teaching responsibilities provide a better educational experience for resident trainees.
